# The Lorenz ratio as a guide to scattering contributions to transport in strongly correlated metals

**DOI:** 10.1073/pnas.2318159121

**Published:** 2024-08-22

**Authors:** Fei Sun, Simli Mishra, Ulrike Stockert, Ramzy Daou, Naoki Kikugawa, Robin S. Perry, Elena Hassinger, Sean A. Hartnoll, Andrew P. Mackenzie, Veronika Sunko

**Affiliations:** ^a^Max Planck Institute for Chemical Physics of Solids, Dresden 01187, Germany; ^b^Laboratoire de Cristallographie et Sciences des Matériaux, Normandie Université, Unité Mixte de Recherche 6508 du CNRS, Ecole Nationale Supérieure d’Ingénieurs de Caen, Université de Caen, Caen 14000, France; ^c^National Institute for Materials Science, Ibaraki 305-0003, Japan; ^d^London Centre for Nanotechnology and Department of Physics and Astronomy, University College London, London WC1E6BT, United Kingdom; ^e^ISIS Neutron and Muon Source, Science and Technology Facilities Council, Didcot OX11 0QX, United Kingdom; ^f^Department of Applied Mathematics and Theoretical Physics, University of Cambridge, Cambridge CB3 0WA, United Kingdom; ^g^School of Physics and Astronomy, University of St. Andrews, St. Andrews KY16 9SS, United Kingdom; ^h^Department of Physics, University of California, Berkeley, CA 94720

**Keywords:** transport in strongly correlated metals, Lorenz ratio, thermal transport, electron–electron scattering

## Abstract

In many physical situations in which many-body assemblies exist at temperature *T*, a characteristic quantum-mechanical time scale of approximately ${\hbar /k}_{B}T$ can be identified in both theory and experiment, leading to speculation that it may be the shortest meaningful time in such circumstances. This behavior can be investigated by probing the scattering rate of electrons in a broad class of materials often referred to as “strongly correlated metals”. It is clear that in some cases only electron–electron scattering can be its cause, while in others it arises from high-temperature scattering of electrons from quantized lattice vibrations, i.e., phonons. In metallic oxides, which are among the most studied materials, analysis of electrical transport does not satisfactorily identify the relevant scattering mechanism at “high” temperatures near room temperature. We therefore employ a contactless optical method to measure thermal diffusivity in two Ru-based layered perovskites, Sr_3_Ru_2_O_7_ and Sr_2_RuO_4_, and use the measurements to extract the dimensionless Lorenz ratio. By comparing our results to the literature data on both conventional and unconventional metals, we show how the analysis of high-temperature thermal transport can both give important insight into dominant scattering mechanisms and be offered as a stringent test of theories attempting to explain anomalous scattering.

## Significance

In many strongly correlated metals, resistivities rise more slowly than the Fermi liquid predictions but continue without saturation to very high temperatures, behavior of interest in condensed matter physics and beyond. For example, observations on the scattering rates of such metals are of relevance for comparison with cold atomic systems and the quark–gluon plasma. An open debate is the scattering mechanism that determines the observed phenomena. Using innovative experimental techniques to probe thermal transport in some representative strongly correlated oxide metals, we show that electron–electron scattering dominates, even at room temperature. This emphasizes potential universalities across different fields and guides the quest to understand potential quantum limits to strong scattering in many-body systems.

The challenge of understanding scattering in systems with very strong interparticle interactions continues to generate interest in many fields of physics. In many different physical situations, a scattering rate $\frac{1}{\tau }≅\frac{k_{B}T}{\hbar }$ can be deduced from the measurement of the appropriate transport coefficients (1–3). To fully account for these experimental findings, plausible mechanisms for both the *T*-linear temperature dependence and its prefactor need to be identified. Although progress has been made, many open questions remain, particularly regarding the reason that the prefactor is so often observed to be close to the simple ratio of the two fundamental constants $k_{B}$ and ℏ.

Study of the transport properties of solids provides an important window on the problem. Indeed, the pronounced *T*-linear d.c. electrical resistivity observed near optimal doping in the copper-oxide high-temperature superconductors has been a cause of fascination for over thirty years (4). Although other experiments such as optical conductivity and photoemission are in principle better probes of the scattering rate of electrons, d.c. resistivity can be carried out in such a wide range of physical conditions (for example, ranges of temperature, high magnetic fields, hydrostatic and uniaxial pressure) that resistivity studies have been highly influential in establishing the ubiquity of the *T*-linear scattering rate (5–9). Their analysis has highlighted one of the central questions in the field: Is the observed scattering fundamentally associated with equilibration, *i.e.,* with inelastic processes, or not? If it is, fascinating links emerge with bounds deduced on the time rate of growth of quantum chaos in thermal systems (10), and with other deductions made from the application of string theory to condensed matter physics (3). However, a concrete counterexample has been known since the work of Peierls nearly a century ago. In the electron–phonon problem at high temperatures, the scattering of electrons from phonons is quasielastic, and the resistivity results from *T-*linear growth of the scattering cross-section, which does not involve internal equilibration of the electron system per se (11, 12). *T*-linearity is not the only case of unusual metallic behavior in materials with strong correlations. In other instances, the resistivity has a power lower than two but continues rising with a metallic temperature dependence to temperatures well above those at which a standard Boltzmann transport shows the mean free path to be falling to of order the lattice spacing. The broader range of unusual metallic behavior (including but not restricted to *T*-linear systems) has come to be described using the term “strongly correlated metals.”

Several theories for the mechanism yielding strongly correlated metallic behavior have been put forward, some purely electronic (13), others investigating how electron–phonon scattering might be relevant across a much wider range of temperatures and circumstances than had previously been thought (14–17). Motivated on the one hand by this theoretical work, and on the other by experiments showing *T*-linear resistivity down to temperatures below 10 mK (8) where any role of phonons seems implausible, our goal was to try and better understand the relative roles of electron–electron and electron–phonon scattering. To do so, we chose to investigate thermal transport, to which both electrons and phonons contribute. Thermal and electrical transport are not independent, and any theory aiming to explain one should aim to simultaneously explain the other, as has been emphasized in e.g., refs. 18 and 19.

For our main experiment we selected two strongly correlated metals, Sr_3_Ru_2_O_7_ and Sr_2_RuO_4_. In both, Fermi liquid *T*^2^ scattering rates are observed at low temperatures (below 10 K and 30 K respectively) in zero magnetic field. By room temperature, the resistivities are rising less strongly than *T*^2^ and with absolute values that are smaller than those that would be expected from a simple extrapolation of the *T*^2^ low-temperature behavior. Crucially, there is a plausible microscopic theory for the crossover from *T*^2^ resistivity in both materials, based on electron–electron scattering in the proximity of van Hove singularities (20). We wanted to investigate whether the electron–electron scattering remains relevant at high temperatures, above 100 K, or whether the electrical transport in this temperature range is dominated by phonon scattering. Our conclusion will be that phonon conduction contributes significantly to the heat transport over a wide range of temperatures but that only happens because electron–electron scattering is very strong. We further argue that similar logic applies to the thermal transport in cuprates, which is qualitatively and quantitatively similar to that seen in our experiments.

## Results

**Fig. 1.**
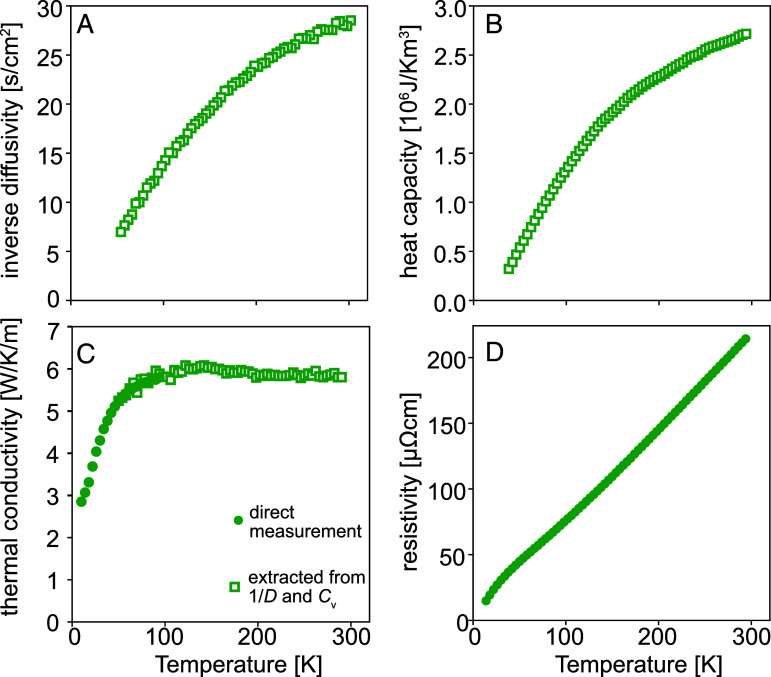
(*A*) Inverse thermal diffusivity of Sr_3_Ru_2_O_7_ measured using the laser-based technique described in the main text. (*B*) Heat capacity of Sr_3_Ru_2_O_7_. (*C*) Thermal conductivity of Sr_3_Ru_2_O_7_ measured at low temperatures with a standard one-heater two-thermometer technique (circles) and extracted from the diffusivity and heat capacity data shown in Fig. 1 *A* and *B* (squares). (*D*) Temperature-dependent resistivity of Sr_3_Ru_2_O_7_, showing the linear behavior above 130 K.

The primary experimental quantity that we set out to measure is the thermal conductivity, which is of particular interest because of evidence from cuprates that it can be decomposed into electron and phonon contributions (21). Although thermal conductivity is widely explored using conventional techniques with heaters and spatially separated thermometers, radiation losses can cause significant systematic errors above 100 K unless particular care is taken to build bespoke apparatus with careful radiation shielding to ensure that the sample and its environment are always at similar temperatures. While we relied on a standard setup for our low temperature measurements, we adopted a different strategy to acquire our high temperature data. We used an optical technique (18, 22) to measure thermal diffusivity *D* (using apparatus described in detail in (23)) and independently measured the heat capacity *c* of the same sample to extract thermal conductivity κ≡cD. Inverse diffusivity and heat capacity data obtained from a Sr_3_Ru_2_O_7_ single crystal, whose growth and characterization are described in (24, 25), are shown in Fig. 1. The two techniques for obtaining κ are nicely complementary, because at the low temperatures where traditional thermal conductivity measurements are most reliable, the precision of optically determined thermal diffusivity becomes poor. We therefore combined the two datasets to yield the data shown in Fig. 1*C* for the thermal conductivity of Sr_3_Ru_2_O_7_ from 5 K to 300 K, with the resistivity shown in Fig. 1*D*.

**Fig. 2.**
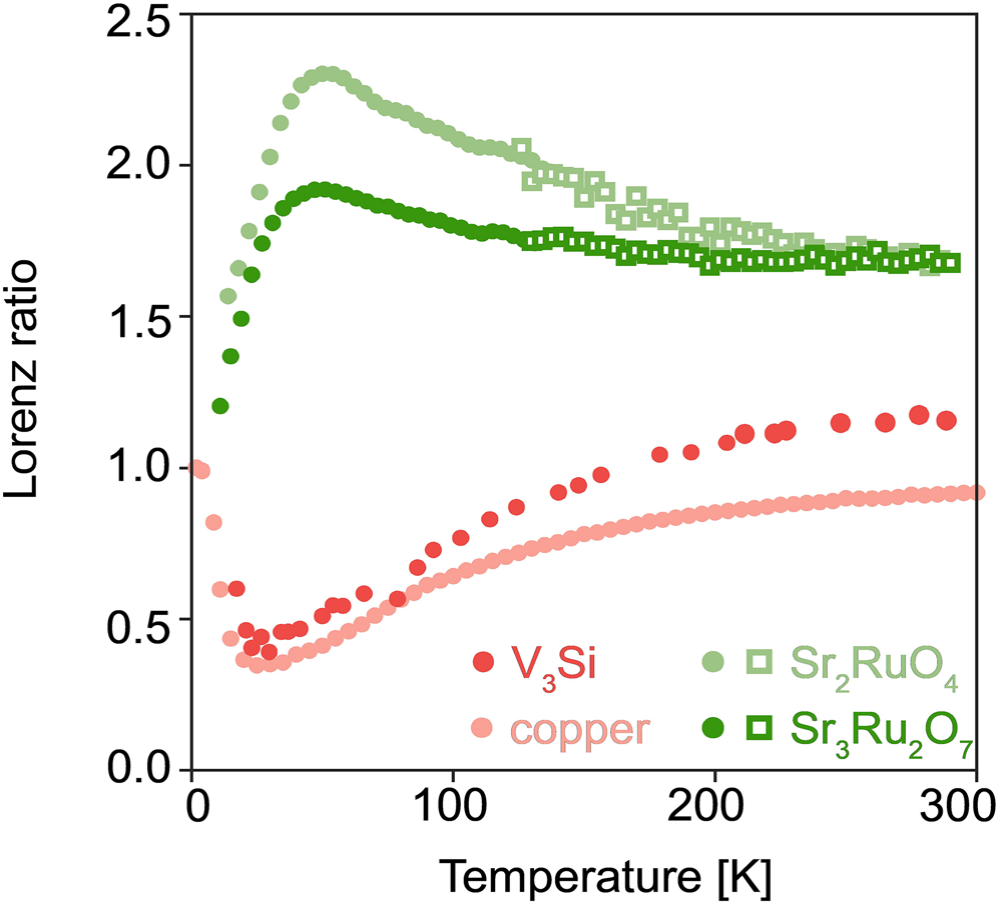
Lorenz ratio for Sr_3_Ru_2_O_7_ (dark green), Sr_2_RuO_4_ (light green), copper (pink; calculated from refs. 32 and 33), and V_3_Si (red; calculated from refs. 34 and 35). Circles and squares refer to the Lorenz ratio calculated from the thermal conductivity obtained from the standard one-heater two-thermometer technique and the diffusivity and heat capacity data, respectively.

In the analysis of thermal conductivity of traditional elemental metals, and indeed many strongly correlated metals in the low-temperature regime, the Lorenz ratio L=κρ/T is an often-quoted quantity [for example (26–29)]. Physically, it is a measure of the relative efficiencies of heat and charge transport. In the zero-temperature limit where the heat and electrical currents are both carried by electrons and the elastic impurity scattering dominates, it famously reduces to the universal Lorenz number (sometimes also referred to as the Sommerfeld value) $L_{0}=\frac{\pi^{2}}{3}{\left(\frac{k_{B}}{e}\right)}^{2}$ = 2.44 × 10^−8^ V^2^K^−2^. The efficiency of thermal transport is therefore conveniently expressed by the dimensionless quantity $L\left(T\right)/L_{0}$. Although $L\left(T\right)/L_{0}$ can be found plotted up to room temperature for traditional metals in many textbooks [e.g., (30)], it has been infrequently plotted over this wide temperature range for correlated electron metals (two examples are refs. 21 and 31). In Fig. 2, we show it for Sr_3_Ru_2_O_7_ and Sr_2_RuO_4_, along with data extracted from literature thermal conductivity and resistivity of Cu, chosen as a representative elemental metal, and V_3_Si. We will start by comparing Sr_3_Ru_2_O_7_ and copper, and return to the other two compounds later in the manuscript.

For both Sr_3_Ru_2_O_7_ and copper $\frac{L\left(T\right)}{L_{0}}≅1$ at low temperatures, as expected. For Cu, the value of $\frac{L\left(T\right)}{L_{0}}$ returns to ≅1 at room temperature but dips far below 1 at intermediate temperature. This finding is typically explained as follows. In elemental metals, the carrier concentration is so high, and the Fermi velocity so large, that electrons completely dominate heat transport over the entire temperature range. Away from the impurity-dominated low temperature limit, the main scattering mechanism is electron–phonon scattering. At intermediate temperatures, phonons do not have sufficient momentum to relax the electron momentum but are able to degrade their energy, so heat is transmitted less efficiently than charge, causing the Lorenz ratio to drop below one. By 300 K, the momentum of a typical phonon is large enough for both the charge and heat currents to be limited by momentum relaxation. The situation resembles that at low temperatures, in the sense that the phonons act like quasi-static scatterers on the characteristic time scale of the electronic motion; in any short-exposure snapshot, the material looks like one at low temperatures with a high level of impurity scattering.

The $L\left(T\right)/L_{0}$ data for Sr_3_Ru_2_O_7_ differ from those in Cu in two key aspects, one qualitative and one quantitative. At a qualitative level, the dip at intermediate temperatures is replaced by a hump, while from a quantitative point of view $L\to L_{0}$ at low temperatures but saturates to a considerably higher value above 200 K. Both observations can be rationalized by postulating that, in Sr_3_Ru_2_O_7_, phonons as well as electrons make a significant contribution to heat transport, as has been stressed in connection with heat transport in the cuprates (14, 18, 21). The hump comes because of the rapid growth in the number of excited phonons, while the high-temperature saturation reflects the saturation of thermal conductivity, and the enhanced value of $\frac{L}{L_{0}}$ is most easily understood in terms of an additive phonon contribution to heat transport. Much less discussion has gone into why the phonon contribution is so visible. Here, we argue that this observation offers information on the relative contributions of electron–electron and electron–phonon scattering. (We note that an analysis presented in ref. 29 suggested that the electronic contribution to L ($L_{\mathrm{el}}$) in Sr_3_Ru_2_O_7_ is equal to $L_{0}$ already at 20 K. However, that conclusion was reached after subtracting a “phonon background” which was not independently determined and was therefore effectively an assumption of the analysis. For that reason, we do not consider the value of $L_{\mathrm{el}}$ to be a settled experimental fact.)

A minimal kinetic framework within which to discuss thermal conductivity in a layered metal, like Sr_3_Ru_2_O_7_, with a quasi-two-dimensional Fermi surface can be summarized with

[1] $$\kappa ={\kappa_{el}+\kappa }_{ph}=\frac{1}{2}c_{el}v_{F}^{2}\tau_{el}+\frac{1}{3}c_{ph}v_{s}^{2}\tau_{ph},$$

where the subscript “el” refers to electrons, subscript “ph” to phonons, $c_{el}$ and $c_{ph}$ are the electronic and phononic contributions to the heat capacity, $v_{F}$ is the average Fermi velocity, $v_{s}$ is the average sound velocity, and the factors of 1/2 and 1/3 appear because the electron (phonon) systems are two- (three-) dimensional, respectively. The expression is based on a quasiparticle picture, but experiments on insulating and metallic cuprates have shown that it is a good starting point for analysis of thermal transport (21, 31); see also *SI Appendix*. Unlike the resistivity, the value and form of κ, and thus of $\frac{L}{L_{0}}$, relies not just on the absolute value of $\tau_{el}$ but on the dimensionless ratio $\tau_{el}/\tau_{ph}$. The data indicate that, above 50 K, the phonon contribution to κ (and hence to *L*) must be approximately as large as the electron one. From inspection of Eq. **1**, $\kappa_{ph}≅\kappa_{el}$ implies that

[2] $$\tau_{\mathrm{ph}}≅\frac{3}{2}\frac{c_{\mathrm{el}}}{c_{\mathrm{ph}}}{\left(\frac{v_{F}}{v_{s}}\right)}^{2}\tau_{\mathrm{el}}.$$

All the relevant parameters in Eq. **1** are known with good accuracy in Sr_3_Ru_2_O_7_, within the approximations inherent to a quasiparticle analysis of multiband systems (2), allowing a numerical estimate of condition [**2**]. This is done, for Sr_3_Ru_2_O_7_ and a number of other materials, in *SI Appendix*. In traditional metals, like Cu, the Fermi velocity is so large that the scattering rate for electrons would need to be 2,500 times larger than that for phonons for the phonon thermal conductivity to dominate. This is clearly unattainable, confirming that the thermal conductivity of traditional metals is electron-dominated. However, both for ruthenates and cuprates it is sufficient for the scattering rate for electrons to be approximately a factor of 40 to 50 higher than that for phonons at room temperature to explain the observed phonon contribution to thermal transport. While the observations of large phonon contributions confirm that $\frac{\tau_{ph}}{\tau_{el}}\approx 40-50$ is achievable, they do not immediately reveal whether this is expected in any material with considerable electron–phonon scattering, or the whether the observation of $\frac{\tau_{ph}}{\tau_{el}}\approx 40-50$ contains additional information about the electronic scattering. We analyze this question below, and confirm that the latter is true.

To examine the thermal conductivity contributions quantitatively, it is necessary to consider the way that scattering rates add. Expressions for the scattering rates of electrons and phonons in a system with electron–electron, electron–phonon, phonon–electron, and anharmonic phonon-phonon scattering are

[3] $$\frac{1}{\tau_{el}}≅\frac{1}{\tau_{el-el}}+\frac{1}{\tau_{el-ph}},\frac{1}{\tau_{ph}}≅\frac{1}{\tau_{ph-el}}+\frac{1}{\tau_{ph-ph}},$$

respectively, (with scattering rates adding in both cases because these are separate scattering mechanisms; disorder scattering is neglected since it is temperature independent and small in these high-purity materials). It is important to note that $\tau_{el-ph}$ and $\tau_{ph-el}$ are not the same; we return to this point later. We have:

[4] $$\frac{\Gamma_{el}}{\Gamma_{ph}}=\frac{\Gamma_{el-el}+\Gamma_{el-ph}}{\Gamma_{ph-el}+\Gamma_{ph-ph}}\geq 50,$$

with Γ=1/τ. Condition [**4**] could in principle be satisfied in several ways. The first is that the highest scattering rate in the problem is $\Gamma_{el-el}$. The second is that $\Gamma_{el-ph}$ is the dominant scattering rate. $\Gamma_{el-ph}$ is usually larger than $\Gamma_{ph-ph}$ because phonon anharmonicity tends to be weaker than el–ph coupling (36, 37). $\Gamma_{el-ph}$ is also typically larger than $\Gamma_{ph-el}$ because the phase space for a phonon to decay into a particle–hole pair is smaller than the phase space for an electron to emit a phonon (38). Although it is a priori unclear whether a combination of these effects could allow for a factor of 50 in [**4**], this seems unlikely: Both $\Gamma_{ph-el}$ and $\Gamma_{el-ph}$ are proportional to the electron–phonon coupling constant, so increasing the coupling does not make $\Gamma_{el-ph}$ the dominant scattering rate.

One way to establish whether electron–phonon coupling alone could satisfy condition [**4**] would be to perform a detailed calculation of the relevant scattering cross-sections for realistic Fermi surfaces. To the best of our knowledge, such a calculation has not been done yet. For the purposes of this paper, we opted to take a more empirical approach. We identified V_3_Si as the material with the highest room temperature resistivity [approximately 70 μΩcm (34)] in which that resistivity is unambiguously dominated by electron–phonon scattering. Its Fermi velocity of 10^5^ ms^–1^ is a factor of sixteen lower than that of Cu but within a factor of two of that of Sr_3_Ru_2_O_7_ (*SI Appendix*, Table S1), meaning that phonons could dominate the thermal conductivity already for $\frac{\Gamma_{el}}{\Gamma_{ph}}\approx 50$. This places V_3_Si in the same part of parameter space as the ruthenates. Further, its strong el–ph coupling makes it a good candidate for the second scenario outlined above ($\Gamma_{el-ph}$ is the dominant scattering rate).

Thermal conductivity measured up to 300 K in carefully radiation-shielded apparatus is reported in the literature (35). Combining the thermal conductivity and resistivity to calculate $L\left(T\right)/L_{0}$ yields the data shown in Fig. 2: in spite of the fact that its room temperature resistivity is a factor of nearly forty higher than that of copper, the Lorenz ratio data of V_3_Si look qualitatively like those of copper, not those of Sr_3_Ru_2_O_7_. The Lorenz ratio of V_3_Si shows that its thermal conductivity is dominantly electronic, even though its electrons move as slowly as those of most strongly correlated metals. This is a nontrivial observation that strongly suggests that, both qualitatively and quantitatively, a temperature-dependent Lorenz ratio of the form seen in Sr_3_Ru_2_O_7_ cannot result simply from strong electron–phonon coupling: $\Gamma_{el-ph}$ and $\Gamma_{ph-el}$ are simultaneously increased, preventing condition [**4**] from being satisfied. It seems that, in metals, strong electron–electron scattering is necessary to allow for the heat transport to be dominated by phonons. The temperature dependence of the Lorenz ratio is a second important diagnostic for this conclusion. As discussed in more detail in *SI Appendix*, S6, the dip seen in the V_3_Si Lorenz ratio naturally occurs in materials in which electron–phonon and phonon–electron scattering are dominant scattering mechanisms.

**Fig. 3.**
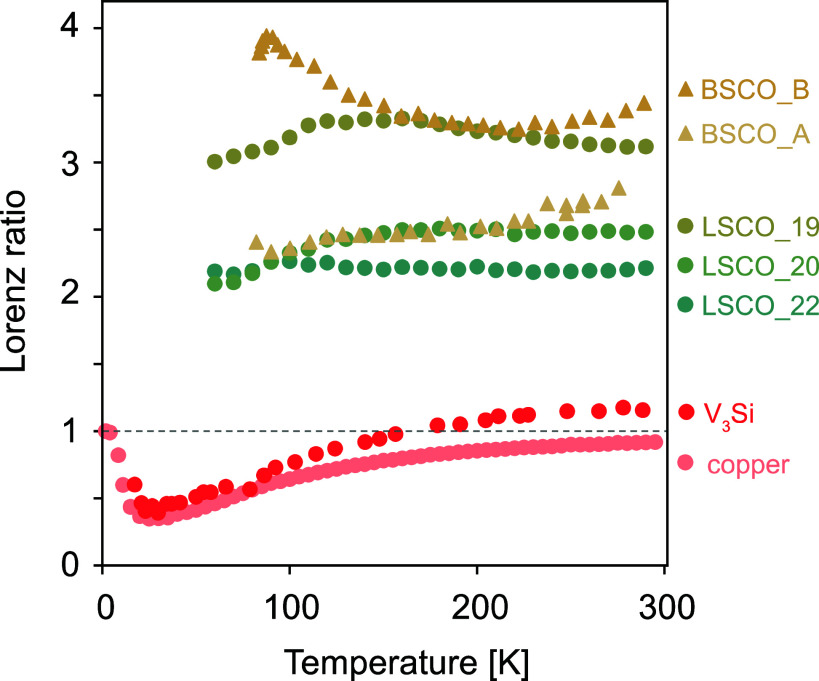
Lorenz ratio of La_2-x_Sr_x_CuO_4_ (x = 0.19, 0.20, 0.22) (39, 40) and two samples (sample A and B) of Bi_2_Sr_2_CaCu_2_O_8_ (41), contrasted with that of V_3_Si and copper.

Although our focus in this paper is Sr_3_Ru_2_O_7_, we wanted to assess the relevance of our findings to other strongly correlated materials. First of all, in Fig. 2, we show that the Lorenz ratio of the ruthenate Sr_2_RuO_4_, measured using a combination of thermal conductivity, thermal diffusivity, and heat capacity measurements, is very similar to that of Sr_3_Ru_2_O_7_. Furthermore, we plot the calculated Lorenz ratio data for La_2-_*_x_*Sr*_x_*CuO_4_ (*x* = 0.19, 0.20, 0.22) (39, 40) and two samples of Bi_2_Sr_2_CaCu_2_O_8_ (41) in Fig. 3, again with the results from copper and V_3_Si. The similarities between cuprates and ruthenates, as well as the difference between those strongly correlated materials and the known electron–phonon scattering dominated materials are very clear.

## Discussion

Figs. 2 and 3 summarize our main empirical result. As we have argued above, as long as electrons and phonons are well-defined quasiparticles, the most plausible explanation for the key differences between the different material classes is strong electron–electron scattering. It is of course possible that this is not a unique explanation and that other scenarios such as the strongly coupled electron–phonon soups of the kind recently proposed in refs. 17 and 18, can also account for the data. *L*/*L*_0_ > 1 can also be obtained in some circumstances within interacting electron models that do not explicitly include phonons (42, 43), although other calculations (27, 44–46), including recent numerical work on the Hubbard model (47, 48) suggested that strong electronic correlations lead to $L/L_{0}<1$, in contrast to the experimental observations. Whatever the final explanation, we hope that we have set a challenge to any theories of scattering in strongly correlated electron materials such as ruthenates and cuprates: matching the qualitative temperature dependences and magnitudes of the dimensionless quantity $L\left(T\right)/L_{0}$ shown in Figs. 2 and 3 should be added to their goals.

If, as we argue is likely, electron–electron scattering is playing a strong role in producing the observed resistivity, including the *T*-linear resistivity in Sr_3_Ru_2_O_7_, this leaves open the possibility that equilibration is relevant to the problem. Our analysis neither relies on microscopic details of the electronic scattering (20, 49, 50) nor gives information on the magnitude of the electronic contribution to $L\left(T\right)/L_{0}$ in the higher temperature region in which it is applied. If the underlying electronic contribution is less than one, processes related to electronic equilibration are relevant.

Another fascinating and so far somewhat sparsely studied aspect of the strongly correlated electron problem is the way in which different types of scattering combine to create the observed resistivities. We believe that our findings show that things are not as simple as assigning standard electron–phonon scattering as the source of much of the *T*-linear resistivity in Sr_3_Ru_2_O_7_ or cuprates: electron–electron processes cannot be ignored. However, that of course does not mean that electron–phonon scattering should be ignored either. It will be interesting to see explicit many-body calculations of the balance between the two such as that recently performed on Sr_2_RuO_4_ (49) and to extend our measurements and analysis to other correlated systems, including strongly overdoped cuprates.

## Materials and Methods

### Sample Preparation.

Single crystals of Sr_3_Ru_2_O_7_ and Sr_2_RuO_4_ were grown in floating zone furnaces as described in the refs. 24 and 51.

### Thermal Diffusivity.

Thermal diffusivity was measured with a spatially resolved optical method described in ref. 23 using two laser beams focused on the sample with radii of approximately 2 μm and separated by a distance *r* of approximately 20 μm. The first laser beam is from an Er-doped fiber ultrafast laser with a wavelength of 780 nm and 80 MHz repetition rate which acts as the source of thermal modulation at a frequency of *ω* ~ 5 kHz determined by passing it through a mechanical chopper. This causes a periodic and local temperature change and the heat diffuses radially at a rate which depends on the thermal diffusivity *D* of the material. The local change in temperature is manifested by a change in temperature-dependent reflectivity. The second beam, He–Ne continuous wave laser with a wavelength of 633 nm, probes this change in reflectivity. A phase lag between the source and the reflected probe beam (*ϕ*) is detected at the frequency (*ω*) and thermal diffusivity can be calculated from the equation:

$$\phi =\sqrt{\frac{r^{2}\omega }{2D}}.$$

This method was used to measure the diffusivity over the broad temperature range of 50 K to 330 K inside a Montana S50 optical cryostat. The sample is mounted on top of a three-dimensional piezoelectric stage with Attocube nanopositioners which gives the freedom to choose the sample position for measurement and adjustment of the focus.

### Heat Capacity.

The heat capacity was measured using a Physical Property Measuring System (PPMS) from Quantum Design at constant pressure. Samples weighing 8.17 mg (Sr_3_Ru_2_O_7_) and 10.23 mg (Sr_2_RuO_4_) were mounted using Apiezon N grease onto a heat capacity puck which has a heater and thermometer, which was then inserted into the cryostat. Measurements were carried out from 10 K to 300 K under high vacuum conditions. The heat capacity is obtained from the relaxation rate of the cooling after the application of a heat pulse to the sample.

### Thermal Conductivity in PPMS.

Thermal transport was measured in a PPMS using the Thermal Transport Option (TTO) under high vacuum across the temperature range 2 ~ 300 K, with simultaneous four-point measurement of thermal conductivity and electrical resistivity. Typical dimensions were spacing between thermal/voltage contacts ~1 mm, width 0.5 mm, and thickness 0.5 mm. The contacts were made using Dupont 6838 silver paint, cured at 180 °C for about two hours. The agreement of the resistivity measurements with Bruin et al. (5) for Sr_3_Ru_2_O_7_ gives evidence that the geometrical uncertainties in our sample mounting and dimension measurement were <15%. The experimental data from this direct measurement for the temperature range from 10 K to 100 K are shown in Fig. 1*C*, and the data for the whole temperature range are shown in *SI Appendix*, Fig. S3.

### Thermal Conductivity in Bespoke Radiation-Shielded Apparatus.

Thermal conductivity was measured using a standard one-heater, two-thermometer technique in which temperatures were measured using fine wire thermocouples attached to the sample. The thermal current was measured using a calibrated heat pipe in series with the sample to reduce the error associated with thermal radiation. The experimental data from this method are shown in *SI Appendix*, Fig. S3.

## Supplementary Material

Appendix 01 (PDF)

## Data Availability

.txt data have been deposited in Edmond (doi: https://doi.org/10.17617/3.Q0JAOZ) (52). All study data are included in the article and/or *SI Appendix*.
