# Supplementary material for: The Lorenz ratio as a guide to scattering contributions to transport in strongly correlated metals
Source: Proc Natl Acad Sci U S A. 2024 Aug 22;121(35):e2318159121. doi: 10.1073/pnas.2318159121 (PMC11363297; doi:10.1073/pnas.2318159121)
Supplement: Supplementary file 1 — Appendix 01 (PDF) [file pnas.2318159121.sapp.pdf]

Supplementary Information

The Lorenz ratio as a guide to scattering contributions to transport in strongly correlated metals

Fei Sun[1], Simli Mishra[1], Ulrike Stockert[1], Ramzy Daou[2], Naoki Kikugawa[3], Robin S. Perry[4,5], Elena Hassinger[1], Sean A. Hartnoll[6], Andrew P. Mackenzie[1,7], Veronika Sunko[1,8]

[1] *Max Planck Institute for Chemical Physics of Solids, 01187 Dresden, Germany*

[2] *Laboratoire de Cristallographie et Sciences des Matériaux (CRISMAT), Norman die Université, UMR6508 CNRS, ENSICAEN, UNICAEN, 14000 Caen, France*

[3] *National Institute for Materials Science, Ibaraki 305-0003, Japan*

[4] *London Centre for Nanotechnology and Department of Physics and Astronomy, University College London, London WC1E6BT, United Kingdom*

[5] *ISIS Neutron and Muon Source, Science and Technology Facilities Council, Didcot OX11 0QX, United Kingdom*

[6] *Department of Applied Mathematics and Theoretical Physics, University of Cambridge, Cambridge CB3 0WA, UK*

[7] *School of Physics and Astronomy, University of St. Andrews, St. Andrews KY16 9SS, UK*

[8] *Department of Physics, University of California, Berkeley, California 94720, USA*

Content

S1 *T*-dependence of thermal diffusivity and heat capacity

S1.1 Thermal Diffusivity

S1.2 Heat Capacity

S2 Comparison between two methods of measuring thermal conductivity

S3 Calculation of the Lorentz ratio for V_3Si from literature data

S4 Comparison of electron and phonon parameters of ruthenates, cuprates and known electron-phonon scattering metals

S5 Comments on the quasiparticle-based decomposition of electron and phonon contributions to thermal conductivity

S6 Statement and analysis of a contrary viewpoint

References

S1 *T*-dependence of thermal diffusivity and heat capacity

S1.1 Thermal Diffusivity

As described in the *Materials and Methods* section, the thermal diffusivity was measured with a spatially resolved optical method [1]. Here we show the temperature-dependent inverse diffusivity for both $Sr_3Ru_2O_7$ and Sr_2RuO_4 in Fig. S1. Note that below 50 K, φ becomes very small and the signal-to-noise ratio falls below useful levels, so data from that temperature range were not used.

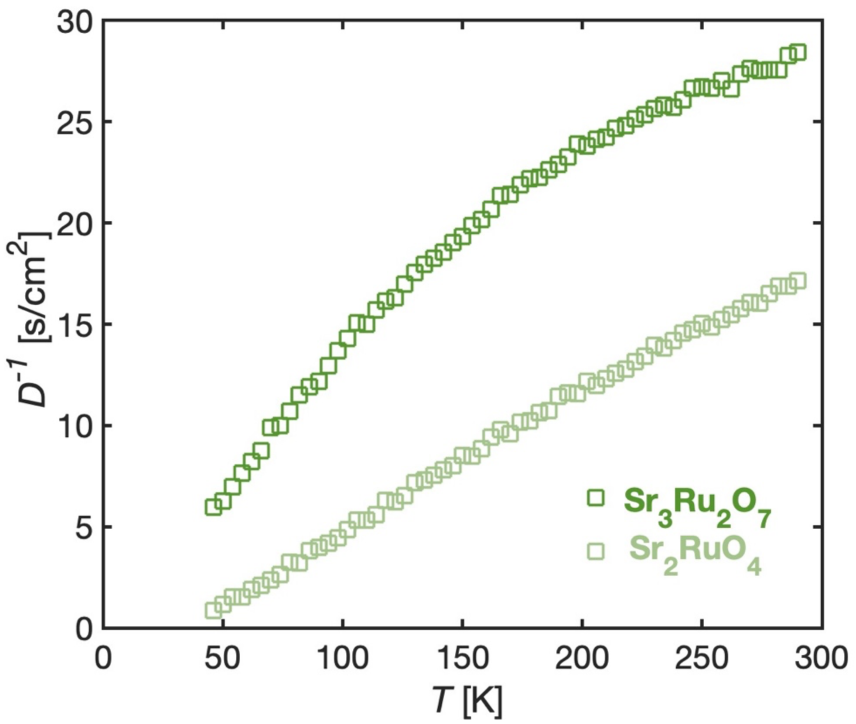

Fig. S1: D^{-1} of $Sr_3Ru_2O_7$ and Sr_2RuO_4 as a function of temperature measured with the optical setup.

S1.2 Heat Capacity

The heat capacity measurements were carried out from 10 K to 300 K under high vacuum conditions. The value of heat capacity is obtained from the relaxation rate of the cooling after the application of a heat pulse to the sample. In Fig. S2, we show the heat capacity as a function of temperature for both $Sr_3Ru_2O_7$ and Sr_2RuO_4.

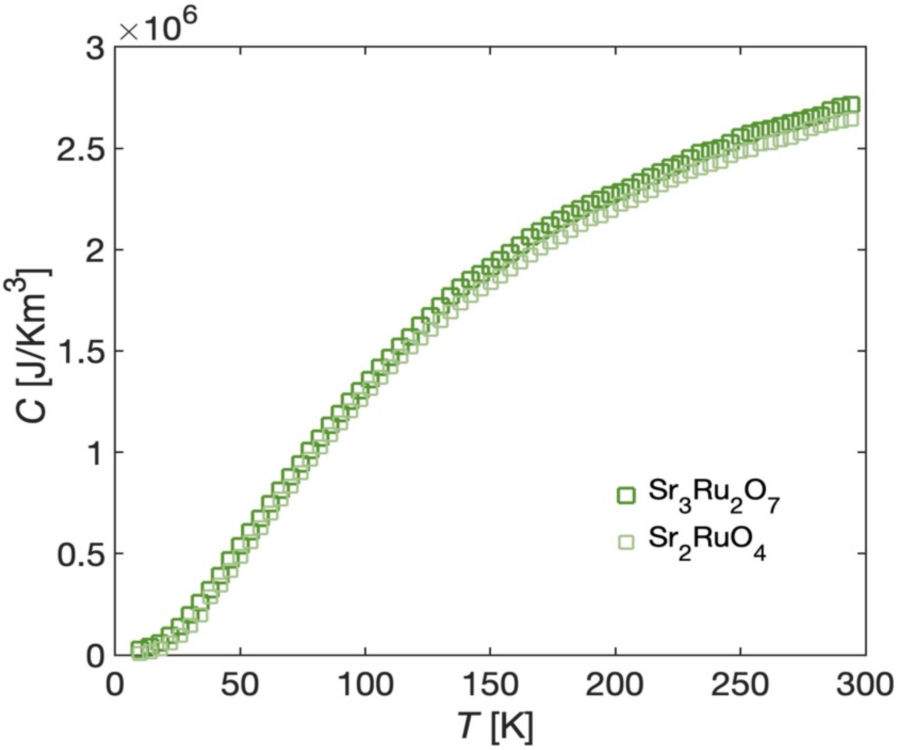

Fig. S2: Heat capacity of $Sr_3Ru_2O_7$ and Sr_2RuO_4 as a function of temperature measured with a PPMS.

S2 Comparison between two methods of measuring thermal conductivity

As discussed in the main text traditional thermal conductivity is more reliable at low temperatures below 100 ~ 120 K and becomes challenging at higher temperatures due to radiation losses. In contrast, the uncertainty in the optical measurement of D^{-1} is more at temperatures typically below 50 K as the phase lag is the smallest here and more susceptible to the presence of experimental offsets. The techniques have an overlapping temperature regime where both values are consistent. κ is calculated from the thermal diffusivity and heat capacity as $\kappa = cD$, where c is volumetric heat capacity. The measured heat capacity c_m is usually in the units of J/(mol Ru K) and can be converted to C through the simple conversion:

$$c\ [\mathrm{J/(K\ m^3)}] = c_\mathrm{m}[\mathrm{J/(mol\ Ru\ K)}]\frac{Z}{N_\mathrm{A}V}$$

where, Z is the number of atoms in formula unit and N_A is the Avogadro's constant and V is the volume of the unit cell. Both traditional thermal conductivity measurements and the optical methods are subject to errors of tens of per cent in the absolute values that they yield, so in order to match the data from the PPMS and optical measurements a scaling factor is necessary. We use a scaling factor of 0.59 for $Sr_3Ru_2O_7$ and 0.65 for Sr_2RuO_4 for the calculated thermal conductivity to obtain the best match in the region around 100 K where both methods are expected to be subject to small systematic errors. This is a single factor, with no attempt made to fit the shape. In $Sr_3Ru_2O_7$ the good match of the temperature dependence between 50 and 150 K gives confidence in the validity of the use of the scaling factor.

In Sr_2RuO_4 the extremely low impurity concentrations lead to very high and somewhat sample-dependent electrical and thermal conductivities below 100 K, and the signal for the optical method is very small. We therefore checked the validity of our high temperature data by direct comparison to the results of the radiation-shielded thermal conductivity performed on a third sample. That crystal was substantially less pure, so the low temperature thermal conductivity shows a much-weakened rise, as expected. However, the optical results agree well with the data from this third sample in the range 100 ~ 300 K that is of primary interest in this paper. (When comparing the two it should be noted that this third sample has its own geometrical uncertainties, and that *no* scale factor was applied to match its data with those from the optical measurements.). The comparison also gives a demonstration of the effects of high temperature

radiation losses on PPMS thermal conductivity results. The rise in the data for both $Sr_3Ru_2O_7$ and Sr_2RuO_4 above approximately 200 K is the result of this source of systematic error.

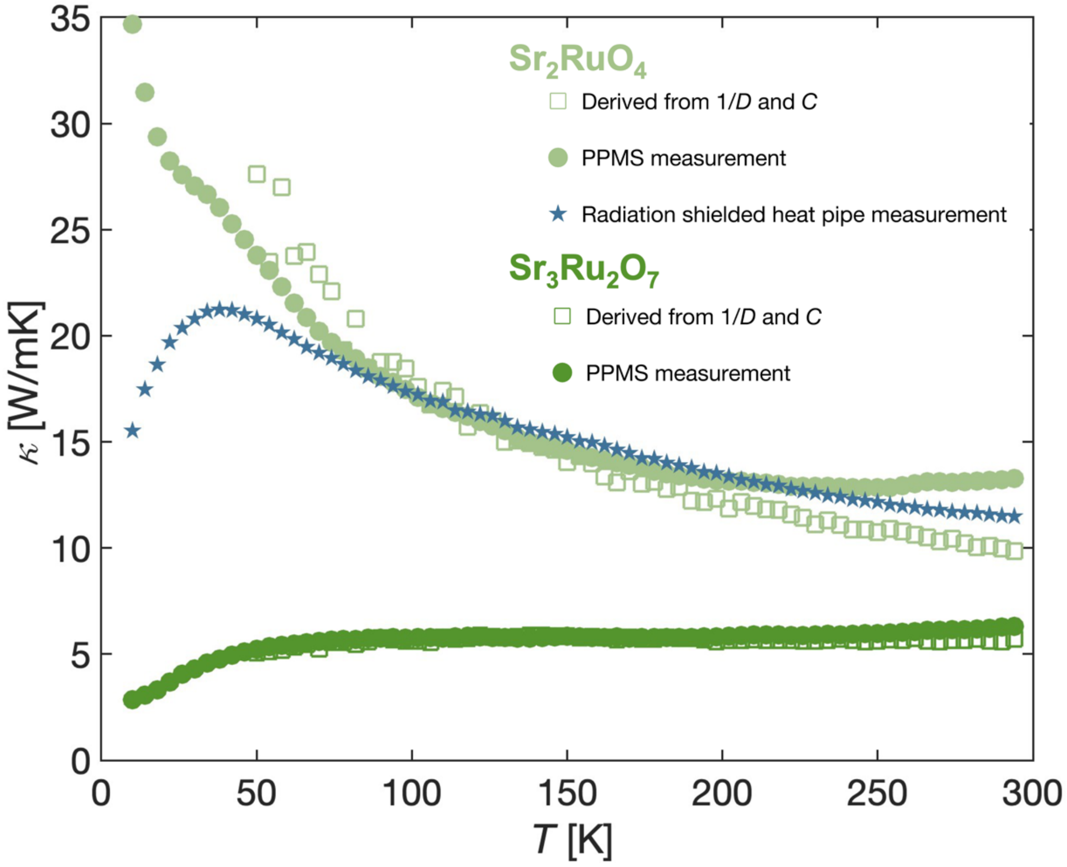

Fig. S3: Thermal conductivity across the full range of temperature from direct measurement and from optical measurement of thermal diffusivity for $Sr_3Ru_2O_7$ and Sr_2RuO_4.

S3 Calculation of the Lorentz ratio for V_3Si from literature data

Thermal conductivity (κ) data were taken from Gladun et al. in Ref. [2], who performed a detailed study of κ vs. T for a high quality V_3Si crystal with residual resistivity ρ_{res} = 1.1 μΩcm, using specialized equipment incorporating radiation shielding to ensure accurate data above 150 K.

Resistivity (ρ) data were taken from the undoped sample from the work of Caton and Vishwanathan (Ref. [3]), with an interpolation used to smoothly bridge some small gaps in their data, as shown in Fig. S5. Since the sample studied by Caton and Vishwanathan had ρ_{res}= 4.1 μΩcm, we subtracted the temperature-independent value of 3.0 μΩcm from all interpolated data before combining them with the Gladun et al. data to calculate $L = \kappa\rho/T$.

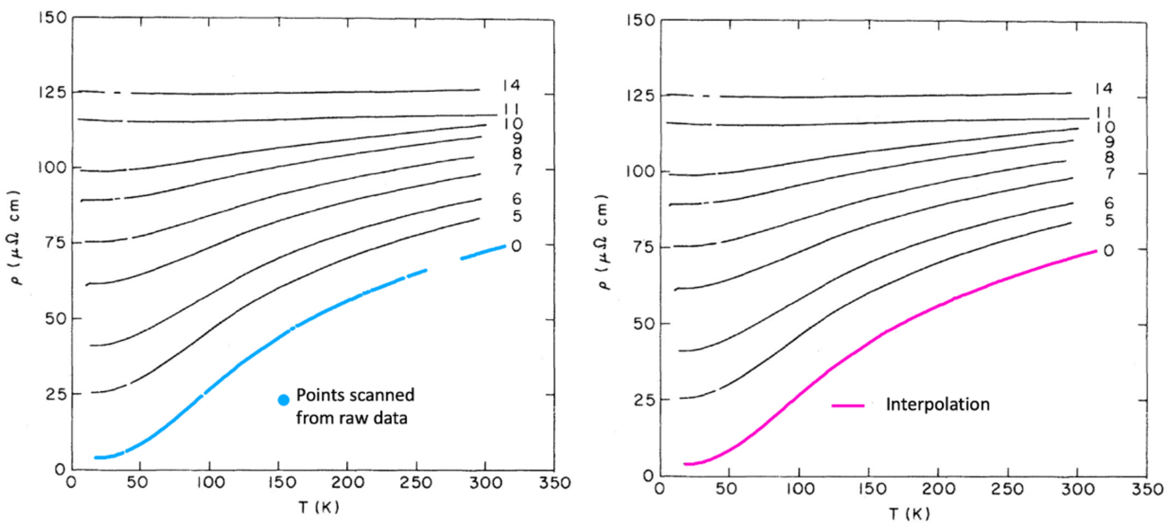

Fig. S4 Scanned data points (blue) and interpolation function (pink) showing the method used to extract resistivity data from Ref. [3].

S4 Comparison of electron and phonon parameters of ruthenates, cuprates and known electron-phonon scattering metals

In this section we give details of the comparisons discussed in the main text between ruthenates, cuprates and two materials, Cu and V_3Si, in which electron-phonon processes are thought to dominate the electronic scattering. As outlined in the main text, the quantity, $\frac{3}{d}\frac{c_{\mathrm{el}}}{c_{\mathrm{ph}}}\left(\frac{v_{\mathrm{F}}}{v_{\mathrm{s}}}\right)^2$, is a measure of the factor by which the total phonon scattering rate must be lower than the total electron scattering rate for phonon and electron contributions to the thermal conductivity to be equal in magnitude. We comment on each column in turn. The parameter d refers to the dimensionality of the electronic system in each material; for every material we assume that the phonon spectrum is best described as being three-dimensional. All data in the remaining columns are at 300 K. To calculate $c_{\mathrm{el}}/c_{\mathrm{ph}}$ we obtain the electronic specific heat coefficient γ, either from the literature or our own measurements, and multiply it by 300 K to estimate the electronic part c_{el} of the total specific heat c_{tot}, which we again obtain either from the literature or our own measurements. The phonon specific heat is then calculated as $c_{\mathrm{ph}} = c_{\mathrm{tot}} - c_{\mathrm{el}}$. Fermi velocity estimates are obtained from either angle-resolved photoemission data or analysis of de Haas – van Alphen effect data, and assumed to be applicable at room temperature. Sound velocities are obtained from the literature. All sources are cited.

Material	d	$c_{\mathrm{el}}/c_{\mathrm{ph}}$	v_{F} (ms^{-1})	v_s (ms^{-1})	$\frac{3}{d}\frac{c_{\mathrm{el}}}{c_{\mathrm{ph}}}\left(\frac{v_{\mathrm{F}}}{v_{\mathrm{s}}}\right)^2$
$Sr_3Ru_2O_7$	2	0.36 [4]**	4.0×10^4 [5]†	4.7×10^3 [6]	40
Sr_2RuO_4	2	0.09 [7]**	8.5×10^4 [8]†	4.7×10^3 [6]	44
$La_{1.8}Sr_{0.2}CuO_4$	2	0.01 [9] [10]	2.3×10^5 [10]	5.9×10^3 [11]	23
$Bi_2Sr_2CaCu_2O_8$	2	0.01 [12] [13]	2.5×10^5 [13]	4.3×10^3 [14]	51
Cu	3	0.01 [15]	1.6×10^6 [16]	3.2×10^3 [17]	2500
V_3Si	3	0.22 [18]**	1.0×10^5 [19]†	7.0×10^3 [20]	45

*Table 1: Experimental values of parameters needed to obtain the ratio between the electronic and phonon scattering rates for different metals. ** Refers to the cases where the value of c_{tot}(300 K) was taken from our own measurements and γ is used from the cited reference. † Denotes the cases where the values of v_F are deduced from the cited de Haas – van Alphen effect results.*

It is seen that the criterion for observing a significant phonon contribution to the thermal conductivity is so extreme in Cu (chosen as a representative of standard metals) that it will never be reached. This explains why, for those standard metals, both the thermal and electrical conductivity are dominated by electron transport. This need not be the case for the other

materials in the table, as long as the total electron scattering rate can be made significantly stronger than the total phonon rate. The most significant finding, as discussed in the main text, is that the criterion for V_3Si is essentially the same as for the strongly correlated cuprates and ruthenates. However, the actual thermal conductivity data for V_3Si are qualitatively more similar to those of Cu than of the strongly correlated materials, suggesting that in V_3Si, the 'back-action' of strong phonon-electron scattering prevents the condition $\frac{c_{\mathrm{el}}}{c_{\mathrm{ph}}}\left(\frac{v_{\mathrm{F}}}{v_{\mathrm{s}}}\right)^2 = 45$ from being satisfied. This in turn suggests that the reason for the observed thermal conductivity of the ruthenates and cuprates is a large electron scattering rate due to a mechanism that gives no back action to the phonon scattering rate. In a naive picture in which electron-electron and electron-phonon scattering can be separated, this suggests that electron-electron processes dominate in the electron scattering in the cuprates and ruthenates, even at room temperature.

S5 Comments on the quasiparticle-based decomposition of electron and phonon contributions to thermal conductivity

The analysis of thermal conductivity in the main text of this paper is based on equation (1):

$$\kappa = \kappa_{\mathrm{el}} + \kappa_{\mathrm{ph}} = \frac{1}{2} c_{\mathrm{el}} v_{\mathrm{F}}^2 \tau_{\mathrm{el}} + \frac{1}{3} c_{\mathrm{ph}} v_{\mathrm{s}}^2 \tau_{\mathrm{ph}} \tag{1}$$

As stated in the text, use of this minimal kinetic expression implies the existence of quasiparticles, an assumption that invites scrutiny. However, we believe that past analysis of cuprate data shows that it is in fact a reasonable starting point, for reasons we now explain.

The value of the Lorenz number L_0 is straightforwardly derived within the quasiparticle picture:

$$c_{\mathrm{el}} = \frac{\pi}{3} k_{\mathrm{B}}^2 T \frac{k_{\mathrm{F}}}{\hbar v_{\mathrm{F}}} \tag{2}$$

Inserting in the expression for κ_{el} gives

$$\kappa_{\mathrm{el}} = \frac{\pi^2}{3} k_{\mathrm{B}}^2 T \frac{k_{\mathrm{F}}}{h} v_{\mathrm{F}} \tau_{\mathrm{el}} \tag{3}$$

while resistivity

$$\rho_{\mathrm{el}} = \frac{h}{e^2 k_{\mathrm{F}} v_{\mathrm{F}} \tau_{\mathrm{el}}} \tag{4}$$

When the scattering rates for thermal and charge currents are the same, the Lorenz ratio

$$\frac{\kappa_{\mathrm{el}} \rho_{\mathrm{el}}}{T} = \frac{\pi^2}{3} \left(\frac{k_{\mathrm{B}}}{e}\right)^2 = L_0 \tag{5}$$

While the combination of fundamental constants would be obtained within any approach, the prefactor is a consequence of the quasiparticle analysis.

The cuprates gave the opportunity to check both the separability of the thermal conductivity into electron and phonon contributions and the validity of the value of L_0 in experiments combining measurements on insulators with those on their doped, metallic counterparts [21] [22]. In the metals, thermal conductivity was measured directly and compared with the sum of that from the insulator (containing only a phonon contribution) and one calculated from the resistivity using $\kappa_{\mathrm{el}} = L_0 T/\rho_{\mathrm{el}}$. Agreement between the two approaches was good (within approximately 20%).

This is our justification for the use of Eq. (1) as the starting framework for the analysis in the main paper. To the level of accuracy we require, it shows that the quasiparticle-based expressions and the separation of electronic and phononic terms in Eq. (1) give sensible answers in relation to the underdoped cuprates, and there is no reason to suspect that the ruthenates and V_3Si are less conventional.

S6 Statement and analysis of a contrary viewpoint

During the refereeing process of the paper, a viewpoint contrary to the one we have presented in the paper was suggested. The argument is as follows: In $Sr_3Ru_2O_7$, the total thermal conductivity at room temperature is approximately 6 W/K/m (see Fig. 1c of the main manuscript). On the assumption that the Wiedemann-Franz law holds in $Sr_3Ru_2O_7$ at room temperature, this means that the phonon contribution to the thermal conductivity is low (approximately 2.6 W/K/m). The reason for this low value is hypothesized to be strong phonon-electron scattering, which means there must also be strong electron-phonon scattering and that it is likely dominant, *i.e.* the opposite of the conclusion given in the main paper that electron-electron scattering must be dominant.

Since this line of reasoning looks plausible at first sight, and others may be tempted to think along similar lines, we comment on it here. First, we re-emphasise that the validity of the Wiedemann-Franz law is an assumption, rather than an established experimental fact. We therefore do not think it should be automatically assumed in the analysis of thermal transport in strongly correlated metals.

However, let us ignore the above caveat for now, and consider again the postulate that electron-phonon processes dominate the scattering in $Sr_3Ru_2O_7$, focusing on the temperature dependence of the Lorenz ratio, rather than just its value at room temperature. If electron-phonon and phonon-electron processes dominate the scattering, there is expected to be a region of low-to-intermediate temperature when the Lorenz ratio drops below one. The phonons cannot contribute significantly to the thermal current because there are fewer of them than at high temperatures, so the electrons must dominate. Further, there will be a range of temperatures over which the low-q phonons that strongly scatter the electrons degrade the electronic contribution to the thermal current more efficiently than they degrade the electrical current. This is a well-known text-book argument, and one that is more or less impossible to avoid.

This 'electron-phonon coupling dip' in the Lorenz ratio is indeed seen in V_3Si, but it is not seen in either $Sr_3Ru_2O_7$ or Sr_2RuO_4. In contrast, there is a maximum in the Lorenz ratio, which can be simply understood in terms of the phonon thermal conductivity rising because of an increased number of phonons which are not immediately strongly scattered by the conduction

electrons. These considerations demonstrate that both the temperature dependence and absolute values of the Lorenz ratio in strongly correlated metals point to the existence of strong electron-electron scattering as the mechanism that makes the phonon contribution to the thermal conductivity so prominent.

References

[1] F. Sun, S. Mishra, P. H. McGuinness, Z. H. Filipiak, I. Markovic, D. A. Sokolov, N. Kikugawa, J. W. Orenstein, S. A. Hartnoll, A. P. Mackenzie and V. Sunko, "A spatially resolved optical method to measure thermal diffusivity," *Rev Sci Instrum,* 94, 043003, (2023).

[2] A. Gladun, C. Gladun, M. Knorn and H. Vinzelberg, "Thermal conductivity of V_3Si," *Phys. Stat. Sol.,* 68, 301 (1981).

[3] R. Caton and R. Viswanathan, "Analysis of the normal-state resistivity for the neutron-irradiated A15 superconductors V_3Si, Nb_3Pt, and Nb_3Al," *Phys. Rev. B,* 25, 179 (1982).

[4] A. W. Rost, S. A. Grigera, J. A. N. Bruin, R. S. Perry, D. Tian, S. Raghu, S. A. Kivelson and A. P. Mackenzie , "Thermodynamics of phase formation in the quantum critical metal $Sr_3Ru_2O_7$," *Proc. Nat. Acad. Sci.,* 108, 16549 (2011).

[5] J.-F. Mercure, A. W. Rost, E. C. T. O'Farrell, S. K. Goh, R. S. Perry, M. L. Sutherland, S. A. Grigera, R. A. Borzi, P. Gegenwart, A. S. A. S. Gibbs and A. P. Mackenzie, "Quantum oscillations near the metamagnetic transition in $Sr_3Ru_2O_7$," *Phys. Rev. B,* 81, 235103 (2010).

[6] C. Lupien, Ultrasound attenuation in the unconventional superconductor Sr_2RuO_4, PhD thesis, Univ. Toronto, 2002.

[7] A. P. Mackenzie, S.-i. Ikeda, Y. Maeno, T. Fujita, S. R. Julian and G. G. Lonzarich, "The Fermi surface topography of Sr_2RuO_4," *J. Phys. Soc. Jpn.,* 67, 385 (1998).

[8] C. Bergemann, S. R. Julian, A. P. Mackenzie, S. NishiZaki and Y. Maeno, "Detailed topography of the Fermi surface of Sr_2RuO_4," *Phys. Rev. Lett.,* 84, 2662 (2000).

[9] K. Sun, J. H. Cho, F. C. Chou, W. C. Lee, L. L. Miller, D. C. Johnston, Y. Hidaka and T. Murakami, "Heat capacity of single-crystal La_2CuO_4 and polycrystalline $La_{2-x}Sr_xCuO_4$ ($0\leq x\leq 0.20$) from 110 to 600 K," *Phys. Rev. B,* 43, 239 (1991).

[10] T. Yoshida, X. J. Zhou, D. H. Lu, S. Komiya, Y. Ando, H. Eisaki, T. Kakeshita, S. Uchida, Z. Hussain, Z. X. Shen, A. Fujimori and A. Fujimori, "Low-energy electronic structure of the high-Tc cuprates $La_{2-x}Sr_xCuO_4$ studied by angle-resolved photoemission spectroscopy," *J. Phys.: Condens. Matter,* 19, 125209 (2007).

[11] J. L. Sarrao, D. Mandrus, A. Migliori, Z. Fisk, I. Tanaka, H. Kojima, P. C. Canfield and P. D. Kodali, "Complete elastic moduli of $La_{2-x}Sr_xCuO_4$ (x= 0.00 and 0.14) near the tetragonal-orthorhombic structural phase transition," *Phys. Rev. B,* 50, 13125 (1994).

[12] A. Junod, K.-Q. Wang, T. Tsukamoto, G. Triscone, B. Revaz, E. Walker and J. Muller, "Specific heat up to 14 tesla and magnetization of a $Bi_2Sr_2CaCu_2O_8$ single crystal thermodynamics of a 2D superconductor," *Physica C: Superconductivity,* 229, 209 (1994).

[13] J. Mesot, M. R. Norman, H. Ding, M. Randeria, J. C. Campuzano, A. Paramekanti, H. M. Fretwell, A. Kaminski, T. Takeuchi, T. Yokoya, T. Sato, T. Takahashi, T. Mochiku and K. Kadowaki, "Superconducting gap anisotropy and quasiparticle interactions: a doping dependent photoemission study," *Phys. Rev. Lett.,* 83, 840 (1999).

[14] G. A. Saunders, C. Fanggao, L. Jiaqiang, Q. Wang, M. Cankurtaran, E. F. Lambson, P. J. Ford and D. P. Almond, "Anisotropy of the elastic and nonlinear acoustic properties of dense textured $Bi_2Sr_2CaCu_2O_{8+y}$," *Phys. Rev. B,* 49, 9862 (1994).

[15] G. K. White and S. J. Collocott, "Heat capacity of reference materials: Cu and W," *Journal of physical and chemical reference data,* 13, 1251 (1984).

[16] N. W. Ashcroft and N. D. Mermin, Solid State Physics, New York: Saunders College Publishing, 1976.

[17] R. C. Weast, Handbook of Chemistry and Physics, 66ed, CRC Press, Boca Raton, 1985.

[18] C.-C. Huang, A. M. Goldman and L. E. Toth, "Specific heat of a transforming V_3Si crystal," *S. State Commun.,* 33, 581 (1980).

[19] T. Terashima, C. Terakura, S. Uji, H. Aoki, J. S. Qualls, D. Hall, J. S. Brooks and T. Fukase, "de Haas--van Alphen oscillations in the A15 superconductor V_3Si," *Physica B: Condensed Matter,* 294, 393 (2001).

[20] L. R. Testardi and T. B. Bateman, "Lattice instability of high-transition-temperature superconductors. II. Single-crystal V_3Si results," *Phys. Rev.,* 154, 402 (1967).

[21] P. B. Allen, X. Du, L. Mihaly and L. Forro, "Thermal conductivity of insulating $Bi_2Sr_2YCu_2O_8$ and superconducting $Bi_2Sr_2CaCu_2O_8$: Failure of the phonon-gas picture," *Phys. Rev. B,* 49, 9073 (1994).

[22] H. Minami, V. W. Wittorff, E. A. Yelland, J. R. Cooper, C. Chen and J. W. Hodby, "Influence of the pseudogap on the thermal conductivity and the Lorenz number of $YBa_2Cu_3O_x$ above T_c," *Phys. Rev. B,* 68, 220503(R) (2003).
